# E-cigarette: a safe tool or a risk factor for oral cancer? A systematic review

**DOI:** 10.4317/jced.62449

**Published:** 2025-02-01

**Authors:** Gaspare Palaia, Mohamed Mohsen, Daniele Pergolini, Valentina Bartone, Angelo Purrazzella, Umberto Romeo, Antonella Polimeni

**Affiliations:** 1 Department of Oral and Maxillo-Facial Sciences, University of Rome “Sapienza”, Via Caserta, 6, Rome, 00161, Italy

## Abstract

**Background:**

Electronic cigarettes (e-cigarettes) are considered relatively safe, thus tobacco and nicotine delivery products have become popular in the last few years. However, the safety of long-term use of these products on oral health is still questionable. E-cigarettes may have potential risks to oral health that can be demonstrated as cellular damage, genetic instability, and mucosal lesions. This review aims to observe the role of e-cigarettes as a risk factor for oral cancer development.

**Material and Methods:**

This systematic review was conducted following the PRISMA guidelines to provide reliable data on the role of electronic cigarettes as a risk factor for oral cancer development. The research was performed on Pubmed and Scopus by three reviewers from the Oral Pathology Department (Sapienza University of Rome) in May 2024. The search terms included: “e-cigarette”, “oral cancer”, and “risk factor” where 32 articles from PubMed and 75 from Scopus were collected. A total of 12 studies met the eligibility criteria: 6 clinical studies and 6 *in vitro*. All the included studies were subjected to quality assessment and data extraction processes. The risk of bias assessment of *in vitro* studies revealed low or unknown risk. None of the studies had any industrial sponsoring and almost all the papers (90%) had the same methods, 64% measured the cell vitality. The qualitative analysis was done for all the included clinical studies using the RoB assessment tool (MINORS). The range of the total RoB score in the comparative studies was between 12 and 23.

**Results:**

The clinical studies involved a total of 413 participants were also included. Most studies did not specify the age and gender of participants and patients were divided into three main groups based on specific criteria: non-smokers, smokers, and e-cigarette users. These studies highlighted cases of mucosal lesions and genetic instability associated with e-cigarette use.

**Conclusions:**

However, the limited long-term data and conflicting results emphasize the need for a larger number of studies, such as randomized controlled trials and cohort studies, to acquire more data about the safety and risks associated with e-cigarettes.

** Key words:**E-cigarettes, squamous cell carcinoma, oral oncology, smoking.

## Introduction

Electronic cigarettes or e-cigarettes are commonly considered as a safer alternative to traditional smoking. Their use increased exponentially, in the last years, especially among young people and their use became very common (1,2).

E-cigarettes are made of a cartridge filled with an e-liquid, a heating element/atomizer necessary to heat the e-liquid to create a vapor that can be inhaled through a mouthpiece and a rechargeable battery. The e-liquid typically contains humectants and flavorings with or without nicotine; once vapourised by the atomizer, the aerosol provides a sensation similar to tobacco smoking but purportedly without harmful effects (1).

The refill liquid without nicotine of e-cigarettes has been found to contain several chemical compounds such as tobacco alkaloids, tobacco-specific nitrosamines, formaldehyde, acetaldehyde, acrolein, metals, Polycyclic Aromatic Hydrocarbons (PAHs), and propylene glycol or glycerin (2).

The ambiguity of conceptions regarding the safety of e-cigarettes highlights the need to research and obtain dependable evidence to alarm consumers for the conscious use.

There is a common assumption that e-cigarette consumption or “vaping” is safer than conventional cigarette smoking (1). However, it has been reported that the heating process can lead to the generation of new decomposition compounds that may be hazardous.

Reactive Oxygen Species (ROS) is a term used to define a variation of oxidant molecules that differ in properties and biological functions ranging between signaling and causing cell damage (1).

This review examines whether e-cigarettes can contribute to the risk of oral cancer, addressing gaps in research on their long-term safety. In this general perspective, the rationale of this systematic review is based on the research of dependable evidence about the possible risks of e-cigarette use in oral cancer.

The *in vitro* studies used samples from various sources where some of them reported cytotoxicity, metabolic activity alterations, apoptosis, increased Bax expression, ROS production, DNA damage, and changes in inflammatory biomarkers in response to e-cigarette exposure.

Several *in vitro* studies have investigated the risk associated with the use of e-cigarettes by exposing oral cells to e-cigarette liquids or vapors. The collected results showed the tendency of oral cells to develop DNA damage and formation of DNA adducts, oxidative stress, metabolic alterations, changes in inflammatory biomarkers, Bax expression, cytotoxicity, and genotoxicity. Some studies have underlined the additional toxicity related to nicotine and flavor additives. Other studies have reported scarce significant harmful effects from e-cigarette exposure. The contradictory results may be related to certain experimental conditions, including exposure doses, cell types, and e-cigarette brands used (1-3).

Nowadays, few studies have described the oral mucosal sequelae associated with e-cigarettes, notably, because they are relatively new to the market. Most of the studies have investigated the effects of short-term use of e-cigarettes, since the long-term use effects are not known yet. The most common side effect notably associated with e-cigarette use is xerostomia, this fact was reported by a 2014 global questionnaire-based survey of 19,414 e-cigarette users (4-6).

Mucosal conditions such as stomatitis, hairy tongue, and angular cheilitis have been proven to have a statistically significant increase in e-cigarette smoke in association with nicotine. E-cigarette users may also experience an increased incidence of nicotine stomatitis due to the e-liquid, they contain, being vaporized by heat (7,8).

The e-cigarette’s internal lithium-ion battery that overheats during vaping can cause explosive injuries that may lead to tooth breakage, alveolar bone fractures, mass formation hematomas, and ulcers due to trauma (9).

The literature still did not report sufficient reliable data on the long-term effects caused by e-cigarettes also because of their very recent introduction to the market. Nevertheless, some case reports about a possible correlation between e-cigarettes and Oral Squamous Cell Carcinoma (OSCC) have been published.

The aim of the study is to focus on the role of e-cigarettes as a risk factor for cellular damage, genetic instability, and oral cancer.

## Material and Methods

This review was conducted following the PRISMA parameters, “Preferred Reporting Items for Systematic Reviews and Meta-analyses” guidelines. The focus question was: Are e-cigarettes a safe tool or a risk factor for oral cancer? The systematic review was registered in Open Science Framework “OSF” Registration DOI. :https://doi.org/10.17605/OSF.IO/3R9XB. The authors have stated explicitly that there are no conflicts of interest in connection with this article.

This study included only human studies (adults) who exclusively smoke e-cigarettes, traditional smokers, former smokers, and non-smokers. They were examined to analyse the risk of oral cancer. While excluding animal studies, non-cancer oral health issues, e-cigarette explosions, smoking cessation, surveys, Studies with incomplete experimental data, reviews (narrative and/or systematic), abstracts, letters to editors, and paid studies. All the studies had to be written in the English language. Almost all kinds of studies were considered: Randomized Controlled Trials (RCT), and clinical trials.

-Search Strategy

Both PubMed and Scopus databases, in May 2024, were searched thoroughly using the MeSH terms, keywords, and terms related to Oral cancer, Risk factor,and e-cigarette in combination with the Boolean operators “AND” and “OR” ( Table 1).

Furthermore, a manual search was performed on the citation and reference lists of the included studies to identify the non-recalled publications in the initial databases search.

-Study Selection

The screening of the studies was performed in two independent stages by two separate reviewers (M. Mohsen and V. Bartone). In the first stage, both titles and abstracts of the resulting studies were screened independently based on the previously mentioned inclusion and exclusion criteria. In the second stage, the confirmation of the selected articles for the review was performed through a full-text read. In case of disagreement between the two reviewers, the third reviewer (G. Palaia) lead the arbitration and discussion in both stages.

-Extraction and Synthesis of Data

Both data collection and synthesis were implemented by the same two reviewers from each of the eligible studies. The extracted data were the author/year, type and number of samples (including the clinical and histopathological perspectives), number of participants, type of e-cigarette/ traditional smoking, smoking index, main outcomes, and conclusions.

Due to the observed heterogeneousness of the data among the included studies concerning the study design, type of e-cigarette, traditional smoking, type and number of samples, the authors were hindered from carrying out the meta-analysis.

-Assessment of Quality and Bias

Different assessment tools were utilized, according to the type of studies included, for the assessment of the quality and risk of bias (RoB).

Each selected study was subjected to the appropriate tool of assessment and scored independently by two reviewers (M. Mohsen and V. Bartone). Conflicts were resolved through arbitration by a third reviewer (G. Palaia).

Based on a systematic review of *in vitro* studies, in which the authors developed and established then used predefined criteria due to the absence of a standard quality tool and risk of bias tool. Selection, performance, and detection bias were the types of assessed biases in this developed criteria (10). The assessment scores were classified into high and low risk; the score “Risk unknown” was used in case of lack of details to assess the bias.

Clinical studies, the MINORS (“Methodological Index for Non-Randomized Studies”) tool was used; it consisted of 12 methodological items that form the quality assessment tool. Both comparative and non-comparative studies can be assessed by the first 8 items. Whereas the remaining 4 items are applied only to comparative studies (11).

The calculation of the total score should be done at the end of the assessment. Three different scores were used: “0” for not reported, “1” for reported but inadequate, or “2” for reported and adequate. The ideal score for comparative studies was 24 and 16 for non-comparative studies.

## Results

The initial search was generated in the period between 2016 and 2024 on both PubMed, which resulted in a total of 75 and Scopus databases, which resulted in a total of 32 studies.

The number of studies was reduced to a total of 91 studies after the application of the automated tools and the elimination of duplicates and non-English language studies; these studies were subjected to title and abstract screening. The 76 studies met the eligibility criteria after the second stage screening (full text read).

**Figure 1:**
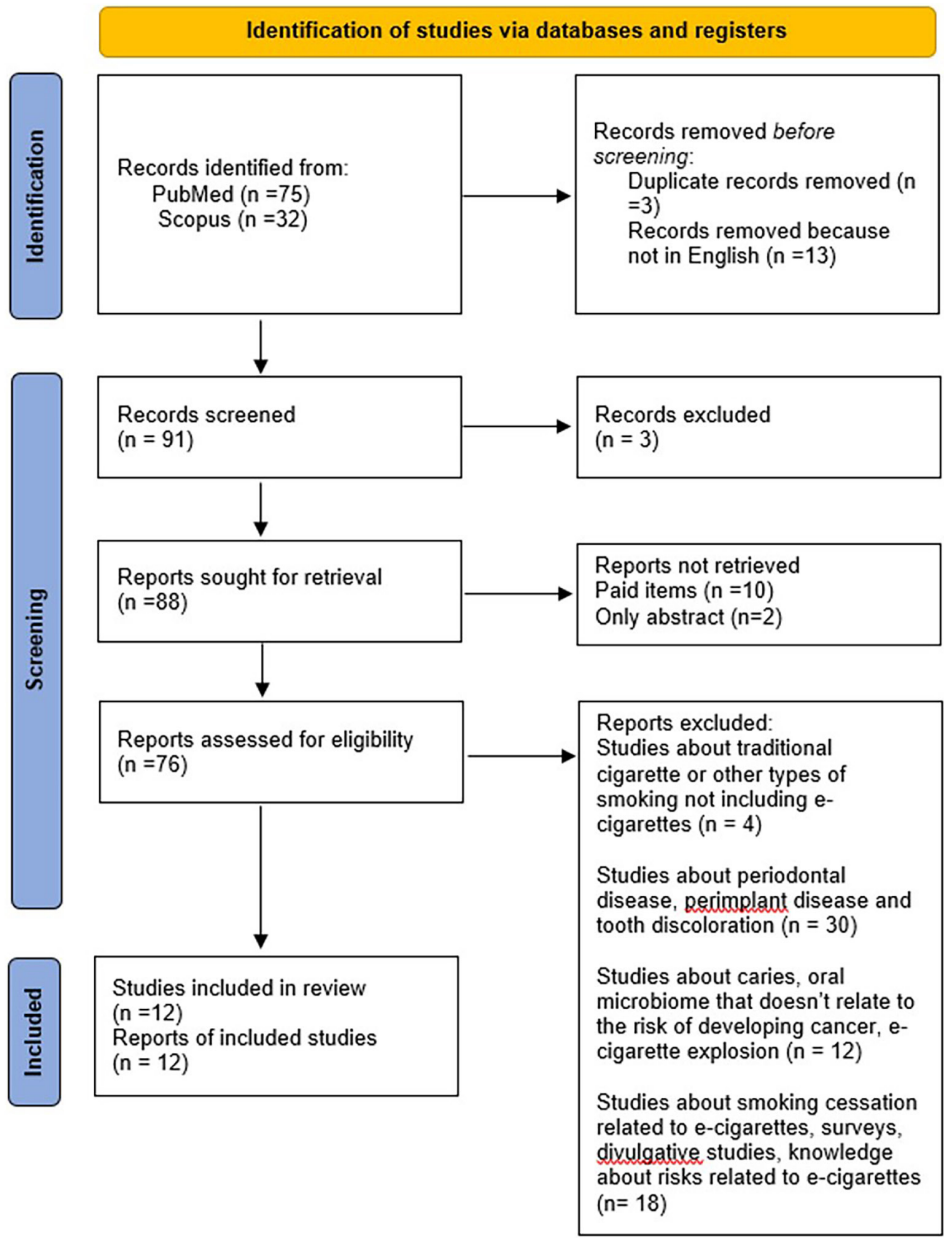
Flow diagram shows the study selection process.

64 studies were excluded after the removal of studies about traditional cigarettes, periodontal diseases, caries and microbiome, and smoking cessation. A total of 12 studies were included in this review and subjected to the extraction of data and quality assessment (Fig. 1) (12).

The included studies were distributed as follows: six clinical and six *in vitro* studies. The risk of bias assessment of *in vitro* studies revealed low or unknown risk. None of the studies had any industrial sponsoring and almost all the papers (90%) had the same methods, 64% measured the cell vitality. On the other hand, they showed a high risk in the randomized exposure, blinded exposure, and sham used for control.

**Figure 2:**
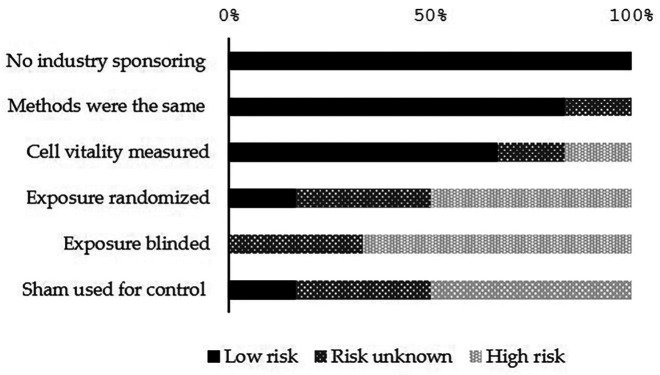
Risk of bias assessment scores in the *in vitro* studies (n=6).

**Figure 3:**
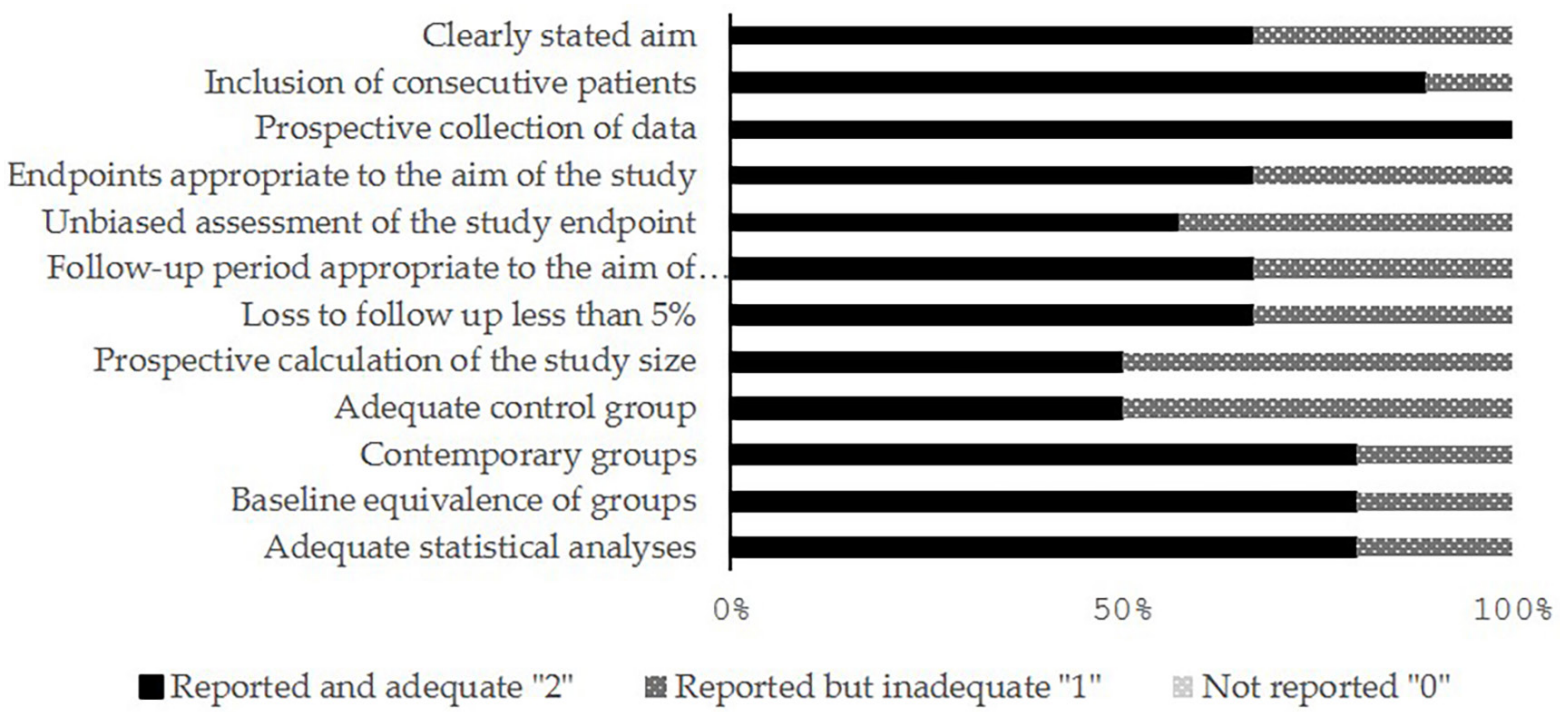
Quality and risk of bias assessment scores of clinical studies (n=6) using the MINORS tool.

The qualitative analysis was done for all the included clinical studies using the RoB assessment tool (MINORS). The range of the total RoB score in the comparative studies was between 12 and 23. Figures 2 and 3 show the scores of different considered domains of the used RoB assessment tools of all the included studies.

According to the inclusion criteria, twelve studies were selected where 413 were participants included. In all the studies there were 144 smokers and 103 e-cigarettes user. While in 6 of the studies, there were 154 non-smokers participants. finally, there were 12 dual smokers participants. An overview of the correlation between e-cigarettes and specific cell alterations is presented in  Table 2, and a comprehensive evaluation of all the included studies in this review is presented in  Table 3 and  Table 4.

In the clinical studies, almost all the articles have not considered participants’ gender and age; and, the participants were categorized into traditional smokers, e-cigarette users, and non-smokers.

The results showed that the exposure to e-cigarette vapor from 24 to 48 hours has caused an alteration in metabolic activity, cytotoxicity, LDH enzyme levels, apoptosis, Bax expression, and Reactive Oxygen Species (ROS) production. ROS production occurs right after 6 hours of exposure and a peak is registered after 24 hours.

Two opposite results were obtained from two different studies about the cotinine levels caused by e-cigarette smoking: Schwarzmeier *et al*. (20) found no correlation. On the other hand, Dongxia *et al*. (13) detected a higher level of cotinine after exposure to eCV.

Three studies have found a positive correlation between e-cigarette vapor and DNA damage: DNA strand breakings, AP sites formation, alteration in gene expression, and TP53 upregulation.

Dongxia *et al*. (13) found a rise in prostaglandin E2 and no effects on Interleukin-1β However, Kamal *et al*. (14) detected higher levels of Interleukin-1β (IL-1β) and Transforming Growth Factor beta (TGF-β) in the e-cigarette consumers group than in the non-smokers group.

-Metabolic Activity

E-cigarette vapor (eCV) altered metabolic activity, increasing it in human gingival fibroblasts after 24 and 48 hours, especially in nicotine-containing e-liquids, which also caused cytotoxic effects (16,23,24).

-Apoptosis

Some studies found that eCV induced apoptosis, marked by an increase in Bax protein and necrosis (3,17). While one study observed no significant effects, with mixed findings on cell death mechanisms (16).

Reactive Oxygen Species (ROS)

E-cigarette vapor led to a significant increase in ROS after 24 hours it reaches a peak and decreases after 48 hours, especially with nicotine-containing vapor (25).

ROS production was linked to eCV’s harmful chemical emissions, such as formaldehyde, heavy metals, diacetyl, carbonyls, and flavoring chemicals (26,27).

-Cotinine

Findings on cotinine, a nicotine metabolite, were inconsistent. Some studies found no correlation between e-cigarette use and cotinine levels while other studies detected significantly higher levels amongst e-cigarette and dual users (20).

-Micronuclei and Cytotoxicity

Exposure to e-cigarette vapors caused the formation of micronuclei and cytotoxicity in oral cells with higher levels of DNA damage and genotoxicity compared to smokers (28,18,19).

-DNA Damage and gene expression

Several studies confirmed DNA strand breaks and alterations in cell cycles after exposure to eCV. Nicotine and flavor additives in e-liquids exacerbated these effects, although some research showed that there was no significant impact (2,17,22).

The adverse cellular responses to e-cigarette aerosols may include proliferation and transition to cancer in some tissue types (25,26). E-cigarette vapor was also found to upregulate genes like TP53, linked to cancer risk, especially in cells exposed to higher puff volumes of eCV (15,17,22).

Recent comparisons of e-cigarette users demonstrate elevated levels of carcinogens compared with controls, as well as the potentially higher risk of transformation of premalignant lesions and development of oral and oesophageal cancers (29,30).

-Salivary Inflammatory Biomarkers

Some studies measured inflammatory biomarkers like prostaglandin E2 and interleukin-1β in saliva finding increased inflammation in e-cigarette users compared to non-smokers, though levels were generally lower than in traditional smokers (13).

-Lactate Dehydrogenase (LDH) Enzyme

LDH levels, which indicate oxidative stress, were significantly higher in smokers and e-cigarette users compared to non-smokers but there was no significant difference observed between smokers and e-cigarette users (21,31).

-Microbiome

E-cigarette use altered oral microbiota, increasing harmful bacteria like Fusobacterium and Prevotella, that are associated with oral cancer. However, a direct link between e-cigarette use and cancer development was not conclusively proven (32).

-Cancer Development

A clinical case reported a young individual who developed aggressive tongue cancer potentially linked to e-cigarette use, though definitive causal evidence is lacking (35).

## Discussion

This study observes the correlation between e-cigarettes and oral cancer. The studies highlighted the complexity of e-cigarettes effects on oral mucosal cells (36).

The e-cigarettes induce changes in the metabolic activity of HGF. This occurs due to prolonged exposure to e-cigarettes, especially in higher nicotine concentrations. This change in metabolic activity occurs after 24 hours of exposure (3).

Signs of cytotoxicity were observed at higher concentrations of nicotine (>2 mg/mL) when the cells became round, translucent, and detached. Genetic instability and cytotoxic effects were more pronounced at higher e-cigarettes liquid concentrations in both normal epithelial and cancerous cells (38).

Apoptosis upregulation can be a consequence of e-cigarettes exposure, especially with nicotine-containing vapor (23).

An overexpression of Bax, a protein related to the process of apoptosis, has occurred after cell exposure to e-cigarettes, suggesting their possible effect on induced cell death (17). However, one of the selected studies reported no significant changes (16).

Oxidative stress is a direct effect of e-cigarettes exposure. An increase in ROS production occurs after only 6 hours, peaking after 24 hours for all the exposed groups compared to the control group (33).

Nicotine-free vapor induced a lower ROS formation, but the effect lasted longer with high ROS levels even after 48 hours. Inconclusive data was collected about the link between cotinine levels and e-cigarettes. Further studies are necessary to understand nicotine metabolism in e-cigarettes users (20).

Tumor suppressor TP53 upregulation is associated with DNA damage (DNA strand breaks and oxidative stress-induced adducts), which suggests possible carcinogenic effects of e-cigarettes. Tumor suppressor TP53 was significantly upregulated in buccal samples (22). Since the studies about DNA damage have a different extent, further studies in standardized exposure conditions are needed to observe the genotoxicity of e-cigarettes (34).

E-cigarettes may cause an increase in Interleukin-1β (IL-1β) and transforming growth factor beta (TGF-β), presenting their inflammatory effects on oral cells (14). However, Dongxia *et al*. found no significant increase in Interleukin-1β (IL-1β). This controversial outcome suggests that more research on e-cigarette-induced inflammatory pathways should be carried out (13).

Schwarzmeier *et al*. found that e-cigarettes group showed a significantly higher number of broken eggs than the smoker group and karyolysis, binucleation, broken egg, and nuclear buds compared to the former smoker and control groups (20).

An increase in E-cadherin levels (in both normal and OSCC cells), B-catenin (in OSCC cells), and Vimentin (in some OSCC cells) suggest that e-cigarettes may cause EMT (37).

LDH is an enzyme released in the presence of damaged cells and its elevation seems to be related to e-cigarettes vapor exposure. In the studies selected, an increase in LDH activity was found in both smokers and e-cigarettes users groups (21).

The present study was associated with a new form of tobacco smoking habit; thus, it has certain limitations, such as the high heterogeneity among the studies, reflecting the lack of standardized study designs. Apoptosis was observed, in some studies, marked by an increase in Bax protein and necrosis (3,17), while one study observed no significant effects. Also, almost all the articles have not considered participants’ gender and age. Opposite results were obtained from various studies that observed the cotinine levels caused by e-cigarettes smoking; according to Schwarzmeier *et al*., no correlation was found (20). On the other hand, a higher level of cotinine was detected after the exposure to e-cigarettes, according to Dongxia *et al*. (13). Some other limitations are the small number of clinical studies, RCT, sample size, cohort mismatch, and limited long-term data.

## Conclusions

E-cigarettes could be considered a risk factor for oral cancer based on the reliable data provided by this review.

However, further studies are needed to investigate e-cigarettes’ long-term effects and their possible adverse effects on metabolic activity, apoptosis, ROS production, DNA integrity, and microbiome composition.

These findings emphasize the need for the cautious use of e-cigarettes and the importance of further scientific investigations.

## Figures and Tables

**Table 1:** The search strategy used in the PubMed and Scopus databases.

Data Base	Search Strategy
PubMed	(("oral cancer" OR oral squamous cell carcinoma OR Oral cavity cancer OR ( "Squamous Cell Carcinoma of Head and Neck/classification"[Mesh] OR "Squamous Cell Carcinoma of Head and Neck/diagnosis"[Mesh] OR "Squamous Cell Carcinoma of Head and Neck/etiology"[Mesh] OR "Squamous Cell Carcinoma of Head and Neck/genetics"[Mesh] OR "Squamous Cell Carcinoma of Head and Neck/pathology"[Mesh] ) OR ( "Mouth Neoplasms/diagnosis"[Mesh] OR "Mouth Neoplasms/etiology"[Mesh] OR "Mouth Neoplasms/genetics"[Mesh] OR "Mouth Neoplasms/pathology"[Mesh] OR "Mouth Neoplasms/prevention and control"[Mesh] )) AND (Risk factor OR Risk Profile OR determinants OR "Risk Factors"[Mesh] OR "Genetic Risk Score"[Mesh] OR Safe OR safety OR "Safety"[Mesh] OR "Chemical Safety"[Mesh])) AND (e-cigarette OR e-cig OR vape OR vape pen OR tanks OR electronic nicotine delivery systems OR "Electronic Nicotine Delivery Systems"[Mesh]) Filters: Humans, English, from 2016 – 2024
Scopus	( TITLE-ABS-KEY ( electronic AND nicotine AND delivery AND systems ) OR TITLE-ABS-KEY ( e-cig* OR e-smoker* OR e-cigarette* OR "electronic cigarette*" ) AND TITLE-ABS-KEY ( stomatognathic AND diseases ) OR TITLE-ABS-KEY ( "risk Factor" ) OR TITLE-ABS-KEY ( "oral health" OR "oral medicine" OR "oral pathology" OR "mouth diseases" OR "periodontal diseases" OR "oral lesions" OR "mucosal lesions" OR tongue OR cheilitis OR stomatitis OR leukoplakia OR teeth OR dental ) OR TITLE-ABS-KEY ( risk AND factor OR risk AND profile OR safety ) OR TITLE-ABS-KEY ( oral AND squamous AND cell AND carcinoma OR oral AND carcinoma ) OR TITLE-ABS-KEY ( smoker OR non AND smoker ) ) AND ( LIMIT-TO ( SUBJAREA , "DENT" ) ) AND ( LIMIT-TO ( DOCTYPE , "ar" ) ) AND ( LIMIT-TO ( EXACTKEYWORD , "Human" ) ) AND ( LIMIT-TO ( LANGUAGE , "English" ) )

**Table 2:** The studies have investigated the correlation between e-cigarettes and specific cell alterations.

Study Topic	Number of studies
Metabolic activity alterations	2
Apoptosis and induced cell death	3
ROS (Reactive Oxygen Species) prodution	3
Cotinine	2
Metanuclear anomalies and cytotoxicity	3
DNA damage and genotoxicity	3
Salivary inflammatory biomarkers	1
LDH (Lactate Dehydrogenase) enzyme	1
Microbiome	1

**Table 3:** Summarizing and evaluation details of all included in vitro studies.

Authors	Sample	Source	Type of Exposure	Main Outcomes	Conclusions
Tsai et al. (2020) (31)	Gingival and tongue squamous cell carcinoma cells	ATCC, Menassas.VA	Green Apple or Red Hot eCig liquid.	Cell invasion, RAGE expression, and Cytokine levels increased	E-cigarette flavoring and nicotine differently impact OSCC invasion and inflammation. It initiates exploration of RAGE-mediated mechanisms in cancer invasion. Identifies molecular pathways used by OSCC for tumor progression.
Vermehrena et al. (2020) (16)	Human gingival fibroblasts	Provitro, Berlin, Germany	24-48 hours exposure to e-cigarette vapor	No cell death detected No immediate harm to HGFs	It can be assumed that e-cig vapor has a less harmful effect when compared to conventional CS.
Sancilio et al. (2015) (3)	Human gingival fibroblasts	Withdrawn during surgical extraction from the retromolar area	24-48 hours exposure to nicotine-containing and nicotine-free e-cig vapor	24 hours: Increased ROS 48 hours: Triggered apoptosis	E-cigarette fluid cytotoxicity on HGFs isn't solely due to nicotine. Further research is needed to clarify e-cig's cytotoxic mechanisms.
Yu V et al. (2015) (17)	Normal epithelial cells as well as head and neck squamous cell carcinoma (HNSCC) cell lines	Withdrawn from from a metastatic lymph node and primary laryngeal tumor	Short- and long-term e-cigarette vapor exposure	Viability: Reduced Apoptosis & Necrosis: Increased DNA Damage: Significant	Study suggests e-cigarettes may be less safe than advertised. E-cig vapors increase DNA damage, cell death, and reduce survival in normal and HNSCC cells, independently of nicotine content. Further research is needed to determine the long-term effects of e-cig.
De Lima et al. (2023) (18)	Normal oral epithelium cell lines, oral squamous cell carcinoma human cell lines, mouse oral cancer cell line	Provided by Dr. Tuula Salo (University of Helsinki).	e-liquid exposure	Proliferation: Promoted Anchorage-Independent Growth: Increased Morphological Changes: Induced Viability: Reduced Flavor Effect: None EMT-Related Gene Expression: altered	E-liquid to induces proliferative and invasive properties along the activation of the EMT process It can contribute to the development of tumorigenesis in normal epithelial cells. It can promote aggressive phenotypes in pre-existing oral malignant cells.
Wisniewskia et al. (2018) (19)	Human-derived dysplastic oral keratinocytes, Human-derived spontaneously immortalized normal oral keratinocytes	DOK; Millipore Sigma, St. Louis, MO.	Nicotine exposure	Cellular Migration: Oral Dysplastic Keratinocytes: Significantly induced Normal Oral Keratinocytes: No effect	First evidence that nicotine increases cell migration in oral dysplastic keratinocytes by activating EGFR signaling through a FASN-dependent mechanism. Nicotine acts as a promoter of malignant progression by activating a pro-oncogenic signaling.

**Table 4:** Summarizing and evaluation details of all included clinical studies.

Authors	Type of the study	Sample	Clinical Exposure	Type of Exposure	Main Outcomes	Conclusions
Schwarzmeier et al. (2020) (20)	Analytical cross sectional	Exfoliative cytology of the lateral region of the tongue and floor of the mouth.	Patients divided into 4 groups: 20 E-cigarette users (with a history of using e-cigarettes for at least 5 months). 22 current smokers of conventional cigarettes. 22 participants who had quit smoking for at least 1 year and no more than 2 years. Control Group: 27 non-smokers.	e-cigarette vapor	Increase in Micronuclei (MN): Induced Cellular Abnormalities: Karyolysis, Karyorrhexis, Binucleation, Broken Eggs, Nuclear Buds	E-cig and alcohol users show damage in oral mucosa cells. Former smokers using both have greater cell damage than non-users.
Pandarathodiyil et al. (2021) (21)	Comparative Analysis	Saliva	Ninety subjects selected and categorized into three groups (controls, n=30, smokers, n=30, and vapers, n=30)	e-cigarette vapor	LDH Activity (mU/ml) showed no significant difference between smokers and vapers.	Findings revealed higher LDH levels in the saliva of vapers versus controls, confirming the cytotoxic effects of e-cigarettes on the oral mucosa.
Guo et al. (2021) (2)	Cohort study	Urine and buccal cells from buccal brushings (cheeks)	30 smokers, 30 e-cigarette users, and 35 nonsmokers	e-cigarette vapor	E-cigarette users had significantly fewer AP sites compared to nonsmokers and smokers.	AP site levels were similar in smokers and non-smokers. E-cigarette users had lower AP sites than both groups. Propylene glycol in e-cig vapor may reduce bacterial-induced inflammation.
Kamal et al. (2022) (14)	Comparative study	Unstimulated whole saliva (uws) sample	150 people Groups: 50 traditional cigarette smokers, 50 electronic cigarette users, 50 non-smoking healthy controls, who had never smoked.	e-cigarette vapor	Increase in IL-1β and TGF-β cytokine	E-cigarette users show higher inflammatory and cancer risk biomarkers than non-smokers, suggesting a lower yet notable risk for systemic diseases compared to traditional cigarettes. The study provides new evidence of e-cigarette harm using a cost-effective, non-invasive approach.
Hamad et al. (2021) (22)	Pilot study	Blood and buccal samples	Three subjects (2M and 1F) Blood and buccal samples were collected from each subject in each visit (total: 18 samples of each patient)	e-cigarette vapor	Buccal samples: upregulation of TP53 linked to puff volume. Blood samples: downregulated MPG affecting DNA repair.	This study shows that vaping 20 puffs significantly affects TP53 expression in human tissues, with vaping behavior being a key factor. A larger study is needed to confirm this relationship.
Ye et al. (2020) (13)	Pilot cross-sectional study	Saliva and gingival crevicular fluid	48 volunteer participants consisting of 4 groups, non-smokers (NS), cigarette smokers (CS), EC and dual EC and cigarette smokers (DS).	e-cigarette vapor	Increase Prostaglandin E2: MPO & MMP-9:, RAGE:Uteroglobi, n/CC-10, and En-RAGE:	Statistically significant differences were observed in oral health biomarkers among different smoking status groups, indicating varying effects of smoking and vaping on oral health.

## Data Availability

The datasets used and/or analyzed during the current study are available from the corresponding author.
